# Medical students’ perceptions and coping strategies during the first wave of the COVID-19 pandemic: studies, clinical implication, and professional identity

**DOI:** 10.1186/s12909-021-03053-4

**Published:** 2021-12-16

**Authors:** Sophie Wurth, Julia Sader, Bernard Cerutti, Barbara Broers, Nadia M. Bajwa, Sebastian Carballo, Monica Escher, Annick Galetto-Lacour, Olivier Grosgurin, Vanessa Lavallard, Georges Savoldelli, Jacques Serratrice, Mathieu Nendaz, Marie-Claude Audétat-Voirol

**Affiliations:** 1 Unit of Development and Research in Medical Education, Faculty of Medicine, University of Geneva, Geneva, Switzerland; 2 Department of General Pediatrics, Children’s Hospital, Geneva University Hospitals, Geneva, Switzerland; 3 Division of General Internal Medicine, Geneva University Hospitals, Geneva, Switzerland; 4 Division of Palliative Medicine, Geneva University Hospitals, Geneva, Switzerland; 5 Division of Emergency Medicine, Geneva University Hospitals, Geneva, Switzerland; 6 Faculty of Medicine, University of Geneva, Geneva, Switzerland; 7 Department of Anaesthesia, Pharmacology and Intensive Care, Geneva University Hospitals, Geneva, Switzerland

**Keywords:** Medical students, COVID-19, Online learning, Perceived stress, Coping strategies, Health care professional identity

## Abstract

**Background:**

The unfolding of the COVID-19 pandemic during spring 2020 has disrupted medical education worldwide. The University of Geneva decided to shift on-site classwork to online learning; many exams were transformed from summative to formative evaluations and most clinical activities were suspended. We aimed to investigate the perceived impact of those adaptations by the students at the Faculty of Medicine.

**Methods:**

We sent an online self-administered survey to medical students from years 2 to 6 of the University of Geneva, three months after the beginning of the pandemic. The survey explored students’ main activities during the first three months of the pandemic, the impact of the crisis on their personal life, on their training and on their professional identity, the level of stress they experienced and which coping strategies they developed. The survey consisted of open-ended and closed questions and was administered in French.

**Results:**

A total of 58.8% of students responded (*n* = 467) and were homogeneously distributed across gender. At the time of the survey, two thirds of the participants were involved in COVID-19-related activities; 72.5% voluntarily participated, mainly fueled by a desire to help and feel useful. Many participants (58.8%) reported a feeling of isolation encountered since the start of the pandemic. Main coping strategies reported were physical activity and increased telecommunications with their loved ones. Most students described a negative impact of the imposed restrictions on their training, reporting decreased motivation and concentration in an unusual or distraction-prone study environment at home and missing interactions with peers and teachers. Students recruited to help at the hospital in the context of increasing staff needs reported a positive impact due to the enriched clinical exposure. Perceived stress levels were manageable across the surveyed population. If changed, the crisis had a largely positive impact on students’ professional identity; most highlighted the importance of the health care profession for society and confirmed their career choice.

**Conclusion:**

Through this comprehensive picture, our study describes the perceived impact of the pandemic on University of Geneva medical students, their training and their professional identity three months after the start of the pandemic. These results allowed us to gain valuable insight that reinforced the relevance of assessing the evolution of the situation in the long run and the importance of developing institutional support tools for medical students throughout their studies.

**Supplementary Information:**

The online version contains supplementary material available at 10.1186/s12909-021-03053-4.

## Background

The rapid development of the corona virus disease (COVID-19) into a pandemic has profoundly disrupted the functioning of the Faculty of Medicine at the University of Geneva since the end of February 2020. Numerous clinical teachers, usually responsible for a fundamental part of the curriculum, were not able to maintain their teaching duties due to urgent needs at the Geneva university hospital. In addition, students throughout the curriculum were seen as a potential work force and got involved in diverse clinical, sanitation or logistic tasks.

Given the measures taken at the university level in the management of the pandemic and with the aim of balancing academic objectives and community-based sanitary needs, the entire teaching framework had to be reorganized at short notice to allow to continue education online. Similarly, faculty had to completely rethink the organization of assessments and exams: with the aim of attesting the acquisition of knowledge and competencies, to stimulate learning and allow the delivery of European Credit Transfer and Accumulation System (ECTS) credits, the Faculty of Medicine set up a mandatory but formative assessment.

The first half of 2020 was hence marked by profound changes in daily and academic life and required the ability to adapt from all stakeholders. In this context, we aimed at understanding how the medical students of the University of Geneva perceived the impact of those adjustments on themselves and consequently on their theoretical and clinical training in order to develop appropriate institutional support strategies to meet the different needs. In addition, the pandemic profoundly affected the medical profession through increasingly strenuous working conditions, required adaptations to help at the forefront and an unprecedented public attention. These circumstances were also likely to affect the student’s vision of the role of a healthcare professional and thus their developing professional identity as this one develops in the context of their studies and with their peers, teachers, and role models [1]. In this context, the Faculty of Medicine of the University of Geneva deemed relevant to draw a global portrait of the perception of this situation by its medical students at different levels within the curriculum (years 2 to 6) with the aim of understanding the impact the crisis had on themselves, their theoretical and clinical training, and on their professional identity.

## Methods

### Design

This study aimed to use a mixed-method design of an exploratory nature to describe medical students’ perceptions and coping strategies during the first wave of the COVID-19 pandemic [2] to describe its perceived impact on themselves, their training, and their professional identity. This mixed method research allows a deeper understanding of an unknown phenomenon through the combination of elements of qualitative and quantitative research approaches [3]. The entire study design is therefore rooted in a phenomenological approach whereby the focus is on the study of the individual’s lived experiences through the exploration of the students’ perceptions of the impact of the pandemic [4]. This was obtained by designing a survey containing on one hand open-ended questions, yielding qualitative data, and on the other hand, Likert type items and Yes-No responses to closed questions in which students were asked to report their perception, yielding the quantitative data.

As this unforeseen context has no precedent in recent decades, the exploratory nature of the design remained descriptive [3, 5].

### Context and data collection

We collected our data via a survey that was carried out through a self-administered online questionnaire using the EvaSys Survey Automation Suite (Electric Paper Informations Systeme GmbH, Lüneburg, Germany). The Faculty of Medicine of Geneva offers a 6-year-long and integrated curriculum. The first year of the curriculum is a year of selection, after which less than one third of the students would be allowed to pass in year 2, and a small percentage be allowed to repeat the year 1 for a second and last attempt. After this selection year, students enter a program including Problem-based learning activities, clinical competence training, and community-based concepts. At the beginning of the 4th year, students enter different clinical settings for an integrated learning of clinical sciences and practice. The 6th year is made of elective clerkships [6, 7]. Given the different organization of the first year of our medical curriculum (selection year) and because the survey was carried out a few weeks before the high stake year 1 exam, a much lower response rate was expected among those students, and thus potentially higher risk of biases. Also, several items were dealing with future professional perspective and identity, and might be not relevant for the population of students in year 1, and thus, other types of questionnaires, not included in the present study, were sent to this class. Individual invitations to participate in the survey were thus sent by email on June 8th 2020 to all medical students of the Faculty of Medicine of Geneva (*N* = 803) from years 2 to 6, with three reminders, three, six, and ten days later. The survey was closed on June 24th 2020 and thus the data collection ended the same day. All the submitted responses were stored in the local database of the faculty EvaSys server.

### Survey content

Medical education requires more than simply acquiring an appropriate level of knowledge and developing relevant skills, it also entails the development of a professional identity. Professional identity formation is key to practice medicine and this process is one that is dynamic and not a fixed concept. As medical students navigate throughout their professional careers in social institutions whether it be at university hospitals, hospices etc., the different experiences they gather make it clearer for themselves who they are in this new identity formation [1, 8]. This process of transformation as they go through life has been described by the social identity theory which suggests that individuals tend to identify themselves as part of a group who they can identify with and who will reinforce they sense of self-esteem [9]. In this context and to address the shared concern about the impact of the pandemic on the medical students of the Faculty of Medicine of the University of Geneva, our research aimed to infer the perceived impact that the pandemic unfolding had on the students’ personal lives, on their training, on their studying and learning as well as on their professional identity [1, 10]. For this, we developed a questionnaire with six main parts. The first part collected demographic information about the participants. The second and third parts focused on the site and type of activities that participants were engaged with during the first three months of the pandemic. A series of items focused on activities directly linked to COVID-19, such as COVID-19 smears, COVID-19 patient admissions at the university hospital or COVID-19 patient follow-up calls among others, and on the motivations for engaging in these activities. The fourth part of the survey collected data on how participants perceived the impact of the crisis on their personal lives and on potential coping strategies they had set up. The fifth section questioned students about the impact of the crisis on their study habits and their perceived learning. One part in this section focused specifically on indicators of stress during that period and was as assessed using the Perceived Stress Scale (PSS), one of the most widely used psychological instruments for measuring the perception of stress [11]. The last part of the survey dealt with the professional identity of students and the impact the crisis had on students’ own perception as future health care professionals. The questionnaire included a total of 75 items that were a mixture of open-ended and closed questions (multiple choice or Likert scale) and was administered in French. The full questionnaire is available in Additional file 1 (original version in French) and in Additional file 2 (translated version in English).

### Data analysis

Analyses of the results were done with open-source statistical computing software R version 3.5.2 (The R Foundation for Statistical Computing, Vienna, Austria). The distribution of the qualitative responses was summarized as percentages of responders or described semi-quantitatively as response groups and their proportions. The distribution of the quantitative variable (PSS) was summarized by the mean and graphically with boxplots. No imputation methods were used to estimate the values of missing responses, expect for the items used to compute the PSS: missing values were replaced by the median of the observations (the percentage of missing values was low and ranged from 1.5 to 2.1% among the ten items used to compute the PSS). Pearson’s Chi-squared test was used to investigate the dependence between categorical variables, and analysis of variance to investigate the difference between different groups of continuous variables.

The analysis of the qualitative data was performed as neutrally as possible to remain descriptive and follow the phenomenological design and showcase the perceived experiences of medical students in the context of a pandemic. We performed a thematic analysis to identify emerging themes [12] . This entailed an active account of the process of analysis, in which our team identified patterns/themes and selected which were of interest and aligned with our research aim [13]. Answers to open-ended questions were imported into the qualitative data analysis software ATLAS.ti (version 8.4.24, ATLAS.ti Scientific Software Development GmbH). Three authors (SW, JS & MCA) analyzed separately which themes emerged before agreeing on themes and subthemes, while maintaining a reflexive stance [14]. The content was then coded accordingly, providing semi-quantitative information about the response groups and their proportions. The obtained data could then be illustrated with corresponding verbatims that were translated from French to English by an English native (JS) and crosschecked by another English native (NB).

The study was granted a waiver from approval by the Ethical Committee of Geneva 465 (Commission Cantonal d’Éthique et de Recherche, CCER). All the authors adhered to the Declaration of Helsinki. Participants were notified about the goals of the study in the invitation e-mail and gave their consent to publish anonymized responses at the end of the study. All data was stored on the servers of the Faculty of medicine at the University of Geneva with access restricted to our research group.

## Results

Our study provides a comprehensive portrait of the perceived impact of the pandemic on the personal and curricular situation of the University of Geneva medical students as well as on their professional identity three months after the start of the pandemic. For each of those aspects, we present quantitative information about response proportions that are then illustrated and supported by qualitative data of the different response groups with appropriate verbatims collected from the open-ended questions. Demographics.

The online self-administered survey was sent to all students from the second to the sixth year of human medicine (*n* = 803) at the Faculty of Medicine at the University of Geneva. Over a period of two weeks (June 8th to 23rd, 2020), a total of 467 students completed the questionnaire (58.2%), homogeneously distributed across gender (60.6% of all female students and 54.4% of all male students enrolled in the curriculum, *p* = 0.085). However, response rates per year of studies differed significantly, ranging between 47.5% (% of 6th year students) and 72.6% (% of 4th year students, *p* = 0.0002).

### Context of the survey – activities during the first three months of the pandemic

Nearly all participants reported a change with respect to planned curriculum activities during the first three months of the pandemic (92.7%). Many students reported participation in clinical activities with patients (44.1%) during those 3 months. Other activities were specified as distance clinical activities (no direct contact to patients, 17.7%), administration (13%) and activities related to research (11.7%). The vast majority of students felt safe in the place where their activities took place (93.5%) and did not feel concerned for their own health (71.8%).

Overall, two thirds of the participants (*n* = 303) were involved in activities directly linked to COVID-19. Of those, nearly three quarters were carried out on a voluntary basis (72.5%). The main motivation for a voluntary involvement in activities linked to COVID-19 was the desire to help and to feel useful.

*“Assist in this crisis, feel useful.”*

Another frequently reported motivation was an interest in and enrichment from participating to this experience from the inside.

*“A desire to partake in the management of the COVID crisis – in the end that’s why I became a doctor. An incredible learning opportunity. “*

A minority of students reported that their participation allowed them to regain a structure to their daily life and avoid social isolation (*n* = 3). Another minority reported remuneration as a motive for voluntary participation (n = 3).

In the context of their activities, students were led to participate in a variety of clinical tasks such as managing patient admissions and follow-up patient care, taking patient histories, vital signs, blood samples and performing swabs and other procedures not limited to patient screening. Other tasks commonly carried out by students were follow-up phone calls with home-based patients diagnosed with COVID-19 and participation in data analysis and research.

Some students also reported the participation in sanitation tasks in the framework of their activities such as disinfection and surface cleaning to comply with the hospital’s prevention program. Others helped with distribution of personal protective equipment in the different hospital departments.

Finally, a variety of logistic and administrative tasks were performed by students, such as data collection and organization, helping patients and staff with paperwork, documenting patient registrations or generating invoices.

### Perceived impact of the COVID-19 pandemic on students and development of coping strategies

We aimed at drawing a comprehensive picture of the impact of the pandemic and its consequences on our students. More than half of the surveyed participants reported a feeling of isolation that had installed over the first three months of the pandemic unfolding (58.8%), which was mainly fuelled by a marked decrease in social contacts and activities. Half of the participants reported the implementation of coping strategies to deal with the changes witnessed over that period of time. The majority indicated physical activity or yoga as a main strategy. Others reported increasing social contacts via phone or video calls or the introduction of a detailed day planning to structure and organize their daily lives.

*“Getting up at a normal time in the morning and filling my day to the fullest, keeping in touch with my friends via messages or phone calls, exercising to clear my head.”*

Alternative strategies described by some students consisted in taking time for oneself, others, or things they did not take time for before and continuing their study program with self-directed learning as much as possible to maintain a certain routine in daily life.

*“Take time for myself, rest and enjoy my loved ones even more.”*

*“Establish a disciplined work routine towards the different subjects in order to stay on track.”*

While most participants referred to the pandemic as a big turmoil of their daily lives, some also described it as a period of personal enrichment or a period of reflexivity.

*“I fell behind in my training but from a human point of view, it was enriching to be able to live this experience and it reinforced my desire to become a doctor.”*

*“I realized that my current training takes an important part of my life, and when it is altered, it is hard to find a work balance and the motivation to go on, the latter being also driven through group learning or clinical activities.”*

### Perceived impact of the COVID-19 pandemic on students’ curriculum

While the curriculum was disrupted, the perceived consequences on students’ training could be separated into two opposite pools. One large group reported the perception of a negative impact of the imposed adjustments, referring to the cancellation of clinical activities and practical coursework and an integral shift of classroom coursework to online learning, which required much more self-directed learning compared to the usual study plan.

*“I did not get the opportunity to train my clinical competencies in medicine. I could not benefit from the clinical activities of the ophtalmology, dermatology, and ENT placements that were cancelled.”*

*“For my training – it got trunkated. Self-based learning through video recordings of the previous year does not replace the interactions that would have happened during problem-based learning sessions, clinical competentcies classes or practical work.”*

A smaller yet consistent group of participants reported an increased exposure to clinical activities due to extraordinary recruitment of students to the hospital and highlighted the positive impact this had on their clinical progression within the curriculum. Those students described the opportunity to get involved in the collective efforts to address the crisis as a highly enriched clinical experience, often increasing motivation.

*“I had the impression that the medical students could be helpful, even bachelor students, that were able to take an active role in the hospital (incredible).”*

*“I learned a lot, gained in autonomy, and I totally felt a sense of belonging at the hospital. I wouldn’t hesitate to do it again.”*

### Perceived impact of the COVID-19 pandemic on studying and learning

Before the pandemic, more than half of the surveyed participants used to study at the university library, while the others mainly worked from home (53% vs. 42% respectively). The majority of participants usually studied alone (71% vs. 29% with one or more persons together). For 95% of the students, the measures enforced by the cantonal authorities to restrict the circulation of the virus imposed working from home during the first three months of the pandemic.

Two thirds of the participants reported that the environment had an impact on their learning abilities. The main issue highlighted by a vast majority of students was a noticeable difficulty to concentrate, mainly caused by increased distractions and a less calm working environment due to noise, the presence of family members or roommates at home and inadequate studying conditions in terms of space or internet connection among other factors.

*“It’s harder to concentrate at home, various distractions, among other things when the rest of the family is at home as well.”*

*“Mainly in terms of concentration. It is harder to concentrate at my place, in a less calm environment with more distractions than in a work environment such as the library.”*

The fact that all teaching was delivered online and that the usual group work and teaching-associated interactions were cancelled decreased motivation for students to a very large extend.

*“I was […] less stimulated, I couldn’t directly ask questions to colleagues or tutors.”*

*“No link with other students at my place (I usually work by myself but always in contact with other students for questions about the learning objectives, organization, …).”*

*“Working from home limits my motivation. I procrastinate much more, which rapidly throws me into a vicious circle of stress and working to catch up: I don’t manage to motivate myself to study, causing me to get stressed and freeze, which again hinders me to work.”*

A related problem that was raised consisted in a sudden equivalence of their work space and their leisure space.

*“Less motivation; the feeling of having no clear cut, of having to work non-stop* ➔ *overload.”*

*“The fact that there is no physical barrier anymore between my study and leisure location was difficult.”*

Most of the students described that the consequences of these circumstances reduced the perceived efficiency and quality of the studies to a large extent.

In addition to this shift to online teaching, most of the exams were moved from a summative to a mandatory but formative evaluation. The majority of students found that this decision had a positive impact on their mental health due to a large reduction in pressure and stress. For many, it even increased their motivation to study the course material since they now studied for themselves rather than for the exams. A small group of students found that the compulsory shift to online learning allowed an improvement in the quality of their studies as they had more time to study. Some also highlighted an increased taking of responsibility for their studies as positive consequence.

*“Much less stress, a much healthier way of learning.”*

*“I took the time to study the coursework I find useful for the profession of a medical doctor and not the coursework that would allow me to pass the exam.”*

“I learned to select the coursework to study instead of blindly swallowing everything under exam pressure.”

However, a large part of the surveyed students reported that the decrease in pressure resulting from the transition of summative to formative assessments decreased their motivation to study, which caused a drop in the quantity of studying and in less diligence during the study periods.

*“I wasn’t as diligent in my studying and the knowledge is clearly not acquired.”*

*“As long as it is not summative, we tend to do the minimum necessary.”*

*“Less commitment to studying because of less pressure.”*

Many students reported that the decrease in the quality and quantity studied caused anxiety about the potential consequences of what they perceived as knowledge gaps for their future careers and the value of their final diploma.

*“I am wondering about the extent of my knowledge gaps from the clinical placements that could not be carried out as usually. And how I will manage to catch up.”*

*“It was a highly enriching experience, but it has probably brought along large gaps in my training. Formative exams, the cancellation of all classwork […] have caused knowledge gaps that will be quite hard to fill and we did not get many tools to overcome them. I think there will be groups that will be less well trained within the same cohort.”*

*“Questions with regard to the validity of the final diploma.”*

A variety of students described feeling abandoned by the faculty in their training, and many reported that they suffered from the cancellation of most small group work and the consequent loss of opportunities to discuss with students and teachers between or after classes.

*“The feeling of being left behind was quite strong, which was what had mostly changed compared to “normal” times.”*

*“An individual training, with little possibility to interact with other students, teachers.”*

**Fig. 1:**
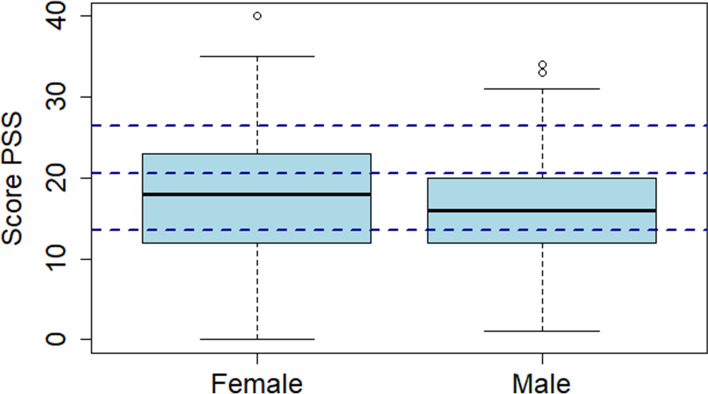
Boxplot of the PSS split by gender (*n* = 294 (female) and 173 (male); p = 0.0001; scores ranging from 0 to 13: low stress, 14–20: moderate stress usually managed, 21–26: moderate stress partially managed, 27–40: high perceived stress)

**Fig. 2:**
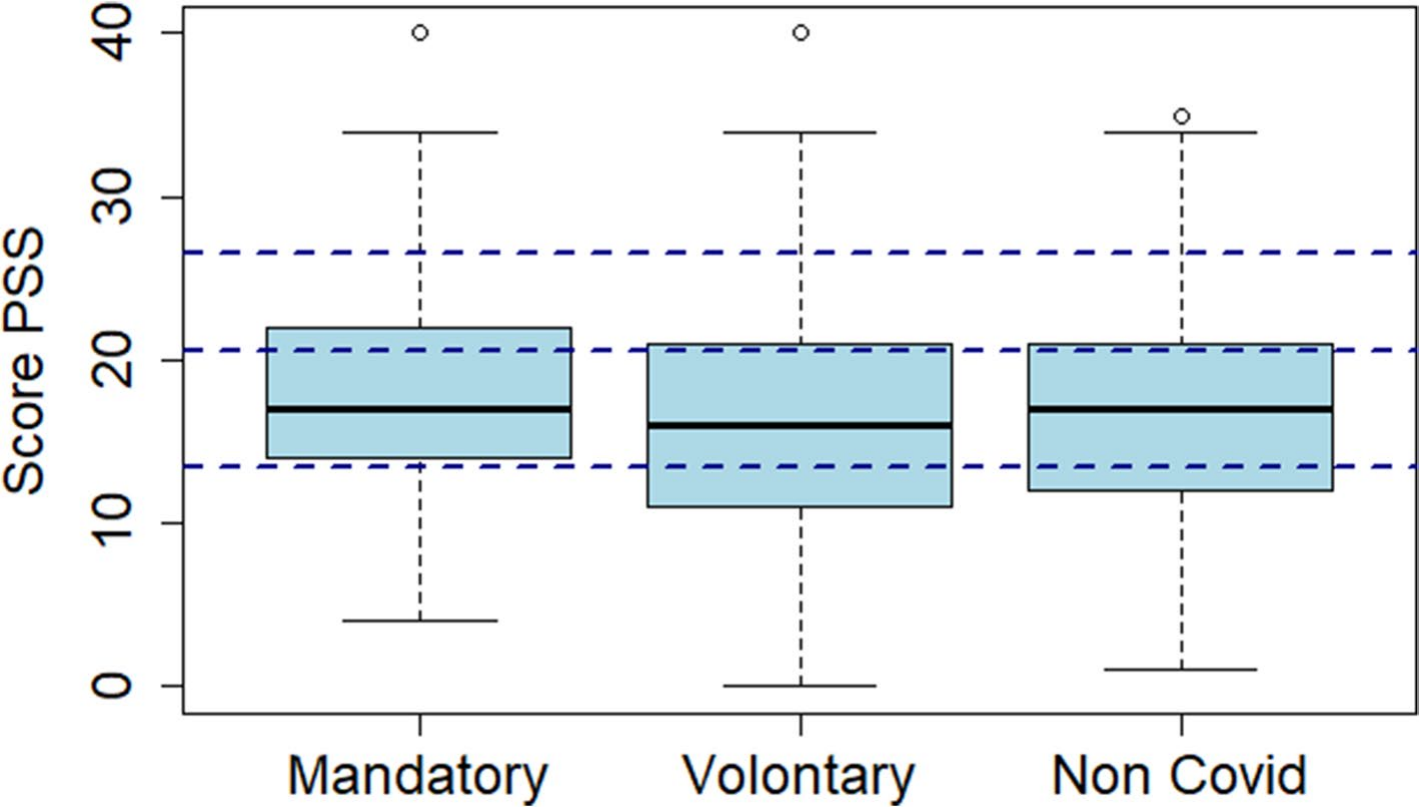
Boxplot of the PSS split by type of activity (*n* = 83 (mandatory COVID), 219 (voluntary COVID), and 152 (non-COVID related); *p* = 0.4170; scores ranging from 0 to 13: low stress, 14–20: moderate stress usually managed, 21–26: moderate stress partially managed, and 27–40: high perceived stress)

We aimed at understanding the level of perceived stress that students experienced during the first three months of the pandemic by evaluating the Perceived Stress scale (PSS). The overall PPS among the responders was 17.1. While we found significant differences between male and female participants on the PSS (15.5 vs 18.1, *p* = 0.0001, Fig. 1), there was no difference by year of studies (*p* value = 0.2040), nor by type of activity the students were engaged with during those three months (*p* = 0.4170, Fig. 2).

### Impact of the COVID-19 pandemic on students’ professional identity

We assessed the impact of the pandemic on the professional identity of our students as future health care professionals. Before the pandemic, the majority of the surveyed participants had already a future specialty in mind (73.4%). Only a minority of students reported a change in this professional perspective after the first three months of the crisis (13.4%). Those students mainly indicated the increased exposure to clinical activities as a positive influence. Some students reported a stronger attraction to a certain specialty, like internal medicine, intensive care, emergency or family medicine.

*“I discovered a new interest for family medicine and for people in precarious situations.”*

*“I aimed at specializing in ophthalmology, but I liked spending a lot of time in internal medicine and I feel that the doctor-patient relationship there is deeper.”*

*“I realized the fundamental importance of being knowledgeable in internal medicine for being able to help during a large sanitary crisis.”*

Others reported that the pandemic raised the issue of a work-life balance.

*“I feel confirmed in my ideas and desire to not neglect time with my family and my loved-ones.”*

*“I am not sure anymore if I am able to take on that role.”*

The vast majority of participants described their role as future health care professionals as supporting and taking care of patients. Other frequently evoked notions being an expert, promoting health and having an impact on the health system. The vision of this role had not changed for the majority of surveyed participants over the first three months of the pandemic (86.2%). For some students, experiencing the pandemic brought afore changes in the vision they had of the role of a health care professional. This change was mostly referred to as positive, driven by a feeling of belonging as well as an increased importance of medical professionals in the eyes of society. Those students reported that they felt confirmed in their career choice.

*“The feeling of belonging to the health care workers and the vision of their commitment.”*

*“The situation allowed me to realize the importance of health care professionals in our society.”*

*“Concerning professional identity, I think the circumstances have confirmed the motivations that drove my choice of going to medical school.”*

Some students described that the pandemic revealed the importance of good interprofessional collaboration and efficient communication with patients in order to be a competent health care provider.

*“Team work and communication are key to good collaboration and patient care.”*

*“I realize the importance of solidarity among health care professionals”.*

*“A lot of kindness and goodwill! The importance of reassuring a patient, of creating trust, of being entirely conscious of potential stereotypes, and how to fight them.”*

In the context of their future profession, the majority of the surveyed students reported that they felt useful during those three months (60.7%) and about one third reported that the feeling of usefulness had evolved over time. Of those indicating an evolution of the feeling of usefulness, most felt more useful and more important for society.

*“I feel reassured to have chosen those studies. I realize that a doctor can do a broad range of things.”*

*“We were able to support science and the population, event before finishing our own studies. This is a strong feeling of usefulness, which was not there beforehand.”*

## Discussion

Disruptions due to the COVID-19 pandemic have compelled medical students and medical schools to adapt since the start of the crisis in March 2020. Major curriculum adjustments have impacted the theoretical and practical training of medical students worldwide with potential consequences on both their mental health and their professional identity [15–18]*.* In this context, we set out to draw a comprehensive picture of the perceived impact that the beginning of this pandemic had on the students, their training and their professional identity at three months into the pandemic.

Our analysis corroborates trends observed in other countries: The majority of our medical students got involved in COVID-19 related activities and did so on a voluntary basis, fuelled by a desire to help. Many reported this unique clinical experience as an enrichment of their training, a perception that has been underlined in other medical schools around the world [19]. On the other side, the adjustments in the curriculum disturbed the usual progression of the students within the program. Most of them reported a decreased concentration and motivation during studying periods, generated by the imposed distance learning in an often less calm and less study-prone environment. The study participants also reported suffering from the missing “normal” interactions with teachers and peers in the usual study plan. Albeit having grown up in a connected and online world, our students reportedly felt that those interactions could not be efficiently replaced by online workshops or virtual group sessions. These insights provide important considerations for curriculum development in general and in the framework of an ongoing pandemic with long-lasting implications on group gatherings in particular. Many students were also concerned about potential gaps in their medical knowledge, and expressed anxiety about the potential consequences. Similar findings have been reported: for instance, a multicentric survey of final year medical students in Belgium found that more than half of the participants felt a qualitative impact on their education and most of those students feared the consequences of the resulting gaps in their training progression [20]. Also, a cross-sectional online survey carried out at US allopathic medical schools found that about one in five medical students was concerned about their future choice of specialty due to a cancellation of most clinical exposure, thereby hindering the exploration of different specialties and the obtention of recommendation letters needed to apply to other programs [21].

Our findings also revealed a variety of positive consequences perceived by a substantial group of participants. Some students realized the importance of certain values and attitudes in medicine, such as interprofessional collaboration and good communication skills to interface effectively with patients. These are fundamentals in the CanMEDs (Canadian Medical Education Directives for Specialists) framework that describes the abilities physicians require to effectively meet the health care needs of the people they serve [22]. Other students reflected on the importance of a healthy work-life balance, which is also key to forming a generation of healthy and efficient medical doctors. The large majority confirmed a feeling of usefulness as future medical doctors that was largely reinforced during the surveyed time. Those positive representations of their role as future doctors relates to the fact that the large majority of participants felt confirmed in their career choice.

Nevertheless, the crisis impact on the study plan and clinical training may have generated anxiety of medical students and might gradually affect their physical, emotional, and mental well-being, especially if this situation is persisting [23]. We compared the stress levels perceived by the study participants during the first three months of the pandemic to stress levels perceived by medical students in “regular” conditions. While our study suggested that our students felt either lower stress [24, 25], similar levels [26], or higher stress [27] when compared to “regular” conditions at medical school, these levels were lower than those that have so far been reported during the COVID-19 outbreak [28]. Those findings are encouraging and support the general impression of students having coped rather well during this first wave. We hypothesize that one of the main reasons for this positive handling consists in many students having taken an active clinical role during the crisis, as highlighted by the high proportion of students who volunteered and felt useful. From early on, the University of Geneva coordinated and organized opportunities to actively involve its medical students in the handling of the COVID-19 crisis. This not only provided a highly enriched extra-curricular learning opportunity but also likely contributed to providing sense and structure to the altered daily life. Indeed, working in the clinical setting during the COVID-19 outbreak has been described as an active coping strategy in terms of psychological well-being in a cohort of undergraduate medical students in France [29]. Another hypothesis for the reasonably good coping with the situation in the first three months of the pandemic by the medical students at the University of Geneva could arise from the local and generally privileged context in Switzerland, where the majority of students are staying with their parents and benefit from a well-functioning social system as well as financial help programs from the university.

### Study strengths and limitations

Nonetheless those valuable insights, our study only provides a transient view of our students’ perception of the impact the crisis had on their lives at three months into the pandemic. At that time, the regular study year was coming close to its end, the country had already endured a six-week nationwide lockdown and the pandemic curve was flattening. Those circumstances are likely to have contributed to a feeling of euphoria that can install at the end of a race. We therefore need to remain vigilant as at this stage; the resilience and energy that are needed to face the pandemic and its consequences come closer to what is required for a marathon.

Our study was able to capture the general feel of the medical students of the University of Geneva after three months into the pandemic. While the satisfactory response rate of the survey and the balanced distribution across gender provided a representative sample for our analyses, the unbalanced response rates between year groups may be a source of bias, especially when we presented the distribution of the responses for the whole sample of responders Another shortcoming consists in potential bias linked to the population of non-responders. While we used a standardized questionnaire to evaluate perceived stress levels to allow comparability with other studies, we did not benefit from an existing “in-house” baseline of experienced stress during medical school.

The timing of our survey allowed us to capture the general feeling after the first three months of the pandemic and retrospectively, in the middle of the crisis. While the time point at which the study was carried out allowed a unique viewpoint of the students’ perception of the impact of the crisis after three months, this single window did not allow us to capture the evolution of the situation beyond the first wave. It remains thus to be seen how students cope with long-lasting disruptions and their consequences. In this framework, the Faculty of Medicine of the University of Geneva has implemented a follow-up and support group for students facing difficulties and continues to offer opportunities for psychological support. The development of such support strategies and efficient alternatives to continue the curriculum in the face of online learning and an overwhelmed clinical environment will therefore be of utmost importance, opening up on future research directions we are taking to understand the long-lasting impact of the pandemic on our students and their training after a year of curriculum adjustments. Finally, while our findings may be difficult to generalize since the study was carried out in a single institution, we believe that the thorough description of the perceived impact of the crisis on our medical students, their training and their developing professional identity can be of interest to other medical faculties as the challenges and adjustments that are being faced by students and faculty alike are similar worldwide.

## Conclusion

Our study captured the perception of the impact the pandemic had on the medical students of the University of Geneva at three months after the start of the crisis. We gained valuable insights into their coping with the imposed adjustments and described how the pandemic affected their theoretical and clinical training as well as their professional identity. While evaluated at a single time point, we highlight the relevance of assessing the evolution of the situation in the long term and underline the importance of developing appropriate institutional support strategies to accompany the students throughout the pandemic and beyond.

## Supplementary Information

**Additional file 1.**

**Additional file 2.**

## Data Availability

The datasets used and analyzed during the current study are available from the corresponding author on reasonable request.

## Abbreviations

COVID-19: Coronavirus disease 19
ECTS: European Credit and Transfer Accumulation System
CanMEDs: Canadian Medical Education Directives for Specialists
PSS: Perceived Stress Score
