# Correction to: Medical students’ perceptions and coping strategies during the first wave of the COVID-19 pandemic: studies, clinical implication, and professional identity

**DOI:** 10.1186/s12909-022-03307-9

**Published:** 2022-04-01

**Authors:** Sophie Wurth, Julia Sader, Bernard Cerutti, Barbara Broers, M. Nadia Bajwa, Sebastian Carballo, Monica Escher, Annick Galetto-Lacour, Olivier Grosgurin, Vanessa Lavallard, Georges Savoldelli, Jacques Serratrice, Mathieu Nendaz, Marie-Claude Audétat-Voiro

**Affiliations:** 1 Unit of Development and Research in Medical Education, Faculty of Medicine, University of Geneva, Geneva, Switzerland; 2 Department of General Pediatrics, Children’s Hospital, Geneva University Hospitals, Geneva, Switzerland; 3 Division of General Internal Medicine, Geneva University Hospitals, Geneva, Switzerland; 4 Division of Palliative Medicine, Geneva University Hospitals, Geneva, Switzerland; 5 Division of Emergency Medicine, Geneva University Hospitals, Geneva, Switzerland; 6 Faculty of Medicine, University of Geneva, Geneva, Switzerland; 7 Department of Anaesthesia, Pharmacology and Intensive Care, Geneva University Hospitals, Geneva, Switzerland

**Correction to: BMC Med Educ 21, 620 (2021)**

**https://doi.org/10.1186/s12909-021-03053-4**

Following publication of the original article [1], we have been informed that the given and last name of each author had been swapped.

The author group has been updated above and original article has been corrected.
