# Supplementary material for: Medical students’ perceptions and coping strategies during the first wave of the COVID-19 pandemic: studies, clinical implication, and professional identity
Source: BMC Med Educ. 2021 Dec 16;21:620. doi: 10.1186/s12909-021-03053-4 (PMC8674407; doi:10.1186/s12909-021-03053-4)
Supplement: Supplementary file 1 — Additional file 1. [file 12909_2021_3053_MOESM1_ESM.pdf]

Cocher : ☐ ☒ ☐ ☐ ☐ Veuillez utiliser un stylo ou un marqueur fin. Ce questionnaire sera traité automatiquement.

Corriger: ☐ ■ ☐ ☒ ☐ Remplissez complètement la case faussement cochée, puis cochez votre nouveau choix.

1. A propos de vous

1.1 Je suis
- ☐ Une femme
- ☐ Un homme
- ☐ Je ne souhaite pas répondre

1.2 Mon année de naissance

1000EDITOR_SUFFIX	☐	☐	☐	☐	☐	☐	☐	☐	☐	☐
100EDITOR_SUFFIX	☐	☐	☐	☐	☐	☐	☐	☐	☐	☐
10EDITOR_SUFFIX	☐	☐	☐	☐	☐	☐	☐	☐	☐	☐
1EDITOR_SUFFIX	☐	☐	☐	☐	☐	☐	☐	☐	☐	☐
	x0	x1	x2	x3	x4	x5	x6	x7	x8	x9

1.3 Je suis actuellement en
- ☐ Bachelor 2
- ☐ Bachelor 3
- ☐ Master 1
- ☐ Master 2
- ☐ Master 3

1.4 Pour les personnes en Master, quels stages avez-vous effectué au cours de ces trois derniers mois ?

2. Activité

2.1 Par rapport à ce qui était prévu dans mon cursus de formation, mon activité a été modifiée au cours de ces trois derniers mois
- ☐ Oui
- ☐ Non

2.2 Au cours des trois derniers mois j'ai eu des activités dans les milieux suivants : (plusieurs réponses possibles)
- ☐ Urgences
- ☐ Soins intensifs - anesthésiologie
- ☐ Soins intermédiaires
- ☐ Médecine interne
- ☐ Médecine de premier recours
- ☐ Infectiologie
- ☐ Autre service clinique
- ☐ Hot-line téléphonique
- ☐ Equipe de recherhce
- ☐ Administration structure hospitalière
- ☐ Cabinet médical - consultation de quartier
- ☐ Bus santé - dépistage
- ☐ Service et administration de santé publique
- ☐ Protection civile
- ☐ Armée
- ☐ ONG
- ☐ Continuité pédagogique (stations formatives etc.)
- ☐ Logistique du bénévolat (centrale + comité)
- ☐ Prévention et contrôle infection
- ☐ Babysitting
- ☐ Aucun des milieux mentionnés ci-joint
- ☐ Autre

2.3 Si vous avez coché "Autre", merci de bien vouloir préciser

2.4 Au cours des trois derniers mois j'ai eu les activités suivantes : (plusieurs réponses possibles)
- ☐ Activités cliniques avec des patients en présentiel
- ☐ Activités cliniques à distance
- ☐ Tâches administratives et de gestion
- ☐ Activités en lien avec la recherche
- ☐ Autres activités

2.5 Si vous avez coché "Autre activités", merci de bien vouloir préciser

2. Activité [suite]

2.6 Au cours des trois derniers mois j'ai eu des activités **principalement** dans le milieu suivant :

- ☐ Urgences
- ☐ Soins intensifs - anesthésiologie
- ☐ Soins intermédiaires
- ☐ Médecine interne
- ☐ Médecine de premier recours
- ☐ Infectiologie
- ☐ Autre service clinique
- ☐ Hot-line téléphonique
- ☐ Equipe de recherche
- ☐ Administration structure hospitalière
- ☐ Cabinet médical - consultation de quartier
- ☐ Bus santé - dépistage
- ☐ Service et administration de santé publique
- ☐ Protection civile
- ☐ Armée
- ☐ ONG
- ☐ Logistique du bénévolat (centrale + comité)
- ☐ Continuité pédagogique (stations formatives, etc.)
- ☐ Prévention et contrôle infection
- ☐ Babysitting
- ☐ Autre

2.7 Si vous avez coché "Autre", merci de bien vouloir préciser

2.8 Au cours des trois derniers mois j'ai eu **principalement** les activités suivantes :

- ☐ Activités cliniques avec des patients en présentiels
- ☐ Activités cliniques à distance
- ☐ Tâches administratives et de gestion
- ☐ Activités en lien avec la recherche
- ☐ Autre activité

2.9 Si vous avez coché "Autre activité", merci de bien vouloir préciser

3. Activités crise Covid-19

3.1 Parmi les activités que vous avez mentionnées dans la section précédente, certaines étaient-elles directement liées à la crise Covid-19 ?

- ☐ Oui
- ☐ Non

3.2 Si oui, ces activités étaient :

- ☐ Volontaires
- ☐ Mandatoires

3.3 Si vous étiez volontaire, quelles ont été vos motivations ?

3.4 Qui a été le plus fréquemment votre superviseur direct pour ces activités directement liées à la crise Covid-19 ?

- ☐ Médecin
- ☐ Infirmier
- ☐ Autre personnel soignant
- ☐ Personnel administratif et de gestion
- ☐ Autre
- ☐ Je n'ai pas l'impression d'avoir été supervisé(e)

3.5 Si "Autre" merci de précisez qui vous a supervisé(e) :

3.6 Quelles sont les tâches cliniques que vous avez effectuées ?

3.7 Quelles sont les tâches sanitaires que vous avez effectuées ?

3. Activités crise Covid-19 [suite]

3.8 Quelles sont les tâches logistiques que vous avez effectuées ?

3.9 Les professionnels de votre lieu de d'activité vous ont-ils offert un soutien psychologique ? ☐ Oui ☐ Non

3.10 Si les professionnels vous ont offert un soutien, merci de préciser :

4. Crise Covid-19

4.1 Avez-vous ressenti de l'isolement au cours de ces trois derniers mois ? ☐ Oui ☐ Non

4.2 Avez-vous mis en place des « coping strategies » (stratégies d'adaptation) pour faire face aux changements de ces derniers mois ? ☐ Oui ☐ Non

4.3 Si vous avez-mis en place des stratégies, lesquelles ? (Décrivez même celles qui vous semblent anodines ou futiles)

4.4 Avez-vous eu le sentiment d'être en sécurité aux cours de ces trois derniers mois sur votre lieu d'activité (stage, étude, etc.) ? ☐ Oui ☐ Non

4.5 Si vous n'avez pas eu ce sentiment merci de préciser pourquoi :

4.6 Avez-vous craint pour votre santé au cours des trois derniers mois ? ☐ Oui ☐ Non

4.7 L'Université de Genève vous a-t-elle offert un soutien psychologique ? ☐ Oui ☐ Non

4.8 Vos enseignants vous ont-ils offert un soutien psychologique ? ☐ Oui ☐ Non

4.9 Si vos enseignants vous ont offert un soutien, merci de préciser :

4.10 A votre avis, qu'est ce qui a le plus changé au cours de ces trois derniers mois pour vous, pour votre formation, et votre image professionnelle ?

5. Mes études

5. Mes études [suite]

5.1 Où étudiez-vous d'habitude la plupart du temps ?
- ☐ Bibliothèque
- ☐ Cafétéria
- ☐ Autre espace au sein de l'université
- ☐ Locaux dans une structure hospitalière (HUG, etc)
- ☐ Mon domicile
- ☐ Autre espace hors université

5.2 La plupart du temps, comment étudiez-vous ?
- ☐ Seul
- ☐ Avec une autre personne
- ☐ En groupe

5.3 Où avez-vous étudié la plupart du temps au cours de ces trois derniers mois ?
- ☐ Bibliothèque
- ☐ Cafétéria
- ☐ Autre espace au sein de l'université
- ☐ Locaux dans une structure hospitalière (HUG, etc)
- ☐ Mon domicile
- ☐ Autre espace hors université

5.4 Estimez vous que l'environnement où vous avez étudié a eu un impact sur votre apprentissage ?
- ☐ Oui
- ☐ Non

5.5 Si cet environnement a eu un impact, à quel niveau ? (Donnez des exemples)

	Pas du tout d'accord	Plutôt pas d'accord	Plutôt d'accord	Tout à fait d'accord
5.6 Depuis le mois de mars la grande majorité des examens a été réorganisée, passant d'une évaluation sanctionnelle à une évaluation formative à caractère obligatoire. Je pense que cela était une bonne décision :	☐	☐	☐	☐

5.7 Quels changements pour vous ont été provoqués par la modification du format d'évaluation ?

Veuillez-vous positionner pour chacune des propositions suivantes :

5. Mes études [suite]

		Beaucoup plus souvent	Plus souvent	Je n'ai pas remarqué de changement	Moins souvent	Beaucoup moins souvent
5.8	Depuis ces trois derniers mois j'ai des difficultés pour dormir	☐	☐	☐	☐	☐
5.9	Depuis ces trois derniers mois je me sens triste	☐	☐	☐	☐	☐
5.10	Depuis ces trois derniers mois je ressens de l'angoisse	☐	☐	☐	☐	☐
5.11	Depuis ces trois derniers mois j'arrive facilement à rester concentré (e)	☐	☐	☐	☐	☐
5.12	Depuis ces trois derniers mois je me sens fatigué (e)	☐	☐	☐	☐	☐
5.13	Depuis ces trois derniers mois je me sens plein d'énergie	☐	☐	☐	☐	☐
5.14	Depuis ces trois derniers mois je me sens motivé (e)	☐	☐	☐	☐	☐
5.15	Depuis ces trois derniers mois j'ai confiance en moi en tant que soignant (e)	☐	☐	☐	☐	☐
5.16	Depuis ces trois derniers mois les relations avec les proches vivant sous le même toit sont difficiles	☐	☐	☐	☐	☐
5.17	Depuis ces trois derniers mois les relations avec mes proches ne vivant pas sous le même toit sont difficiles	☐	☐	☐	☐	☐
5.18	Depuis ces trois derniers mois je pense à des problèmes d'argent me concernant	☐	☐	☐	☐	☐

		Jamais	Presque jamais	Parfois	Assez souvent	Souvent
5.19	Au cours des trois derniers mois combien de fois, avez-vous été dérangé (e) par un évènement inattendu ?	☐	☐	☐	☐	☐
5.20	Au cours des trois derniers mois combien de fois vous a t-il semblé difficile de contrôler les choses importantes de votre vie ?	☐	☐	☐	☐	☐
5.21	Au cours des trois derniers mois combien de fois vous êtes-vous senti(e) nerveux(se) ou stressé(e) ?	☐	☐	☐	☐	☐
5.22	Au cours des trois derniers mois combien de fois vous êtes-vous senti(e) confiant(e) à prendre en main vos problèmes personnels ?	☐	☐	☐	☐	☐
5.23	Au cours des trois derniers mois combien de fois avez-vous senti que les choses allaient comme vous le vouliez ?	☐	☐	☐	☐	☐
5.24	Au cours des trois derniers mois combien de fois avez-vous pensé que vous ne pouviez pas assumer toutes les choses que vous deviez faire ?	☐	☐	☐	☐	☐
5.25	Au cours des trois derniers mois combien de fois avez-vous été capable de maîtriser votre énervement ?	☐	☐	☐	☐	☐
5.26	Au cours des trois derniers mois combien de fois avez-vous senti que vous dominiez la situation ?	☐	☐	☐	☐	☐

5. Mes études [suite]

5.27 Au cours des trois derniers mois combien de fois vous êtes-vous senti(e) irrité(e) parce que des évènements échappaient à votre contrôle ? ☐ ☐ ☐ ☐ ☐

5.28 Au cours des trois derniers mois combien de fois avez-vous trouvé que les difficultés s’accumulaient à un tel point que vous ne pouviez les contrôler ? ☐ ☐ ☐ ☐ ☐

6. Identité professionnelle

6.1 Avez-vous une spécialité en tête concernant votre future carrière professionnelle ? ☐ Oui ☐ Non

6.2 Si oui laquelle ou lesquelles ?

6.3 Ces trois derniers mois ont-ils changé votre perspective professionnelle ? ☐ Oui ☐ Non

6.4 Si oui pourriez-vous décrire cela ?

6.5 Comment définiriez-vous votre rôle en tant que futur médecin ?

6.6 Votre vision de ce rôle a-t-elle changé au cours de ces trois derniers mois ? ☐ Oui ☐ Non

6.7 Si votre vision de ce rôle a changé, pourriez-vous décrire en quoi ?

6.8 Vous êtes vous senti(e) utile au cours de ces trois derniers mois ? ☐ Oui ☐ Non

6.9 Votre sentiment d'utilité en tant que futur médecin a-t-il évolué au cours de ces trois derniers mois ? ☐ Oui ☐ Non

6.10 Si oui, en quoi ce sentiment a-t-il changé ?

7. Pour la suite

7.1 Seriez-vous d’accord de participer à des focus groups ? ☐ Oui ☐ Non

7.2 Seriez-vous d’accord pour que l'on vous recontacte pour participer à des entretiens ? ☐ Oui ☐ Non

7.3 Seriez-vous d’accord pour que l'on vous recontacte dans les années qui viennent pour les suites de cette étude ? ☐ Oui ☐ Non

7. Pour la suite [suite]

7.4 A quelle adresse email ou numéro de téléphone souhaitez-vous être contacté(e) ?

Merci pour votre participation !
