# Supplementary material for: Medical students’ perceptions and coping strategies during the first wave of the COVID-19 pandemic: studies, clinical implication, and professional identity
Source: BMC Med Educ. 2021 Dec 16;21:620. doi: 10.1186/s12909-021-03053-4 (PMC8674407; doi:10.1186/s12909-021-03053-4)
Supplement: Supplementary file 2 — Additional file 2. [file 12909_2021_3053_MOESM2_ESM.pdf]

# Questionnaire [English translation]

- Regular font: closed questions
- **Bold font: open-ended questions**

1. About you
   1. I am
      - A woman
      - A man
      - I wish not to specify
   2. My birth year
      - 1974-1977
      - 1978-1980
      - 1981-1984
      - 1985-1987
      - 1988-1991
      - 1992-1995
      - 1996-1998
      - 1999-2002
   3. I am currently in
      - Bachelor 2
      - Bachelor 3
      - Master 1
      - Master 2
      - Master 3
   4. **For students at the master's level, which clerkship have you been doing in the past three months?**
2. Activity
   1. Compared to what was planned in my training course, my activity has changed over the past three months.
      - Yes
      - No
   2. During the past three months, I carried out activities in the following environments (several answers possible)
      - Emergency
      - Intensive care - anaesthesiology
      - Intermediate care
      - Internal medicine
      - Primary care medicine
      - Infectiology
      - Other clinical service
      - Telephone hotline
      - Research unit
      - Hospital administration
      - Medical office - neighbourhood consultation
      - Geneva Health bus - screening
      - Public health service and administration
      - Civil protection
      - Army
      - NGO
      - Volunteering logistics (central and committee)
      - Pedagogical continuity (OSCE training stations)
      - Prevention and infection control
      - Babysitting
      - Other
   3. **If you checked "Other", please specify**
   4. During the past three months, I carried out the following activities (several answers possible)
      - Clinical activities with face-to-face patients

- Distance clinical activities
- Administrative and management tasks
- Activities linked to research
- Other activities

5. **If you checked "Other activities", thank you for specifying.**
6. During the past three months, I carried out **mainly** the activities in the following environment:
   - Emergency
   - Intensive care - anaesthesiology
   - Intermediate care
   - Internal medicine
   - Primary care medicine
   - Infectiology
   - Other clinical service
   - Telephone hotline
   - Research unit
   - Hospital administration
   - Medical office - neighbourhood consultation
   - Geneva Health bus - screening
   - Public health service and administration
   - Civil protection
   - Army
   - NGO
   - Volunteering logistics (central and committee)
   - Pedagogical continuity (OSCE training stations)
   - Prevention and infection control
   - Babysitting
   - Other
7. **If you checked "Other", thank you for specifying.**
8. During the past three months, I carried out **mainly** the following activities:
   - Clinical activities with face-to-face patients
   - Distance clinical activities
   - Administrative and management tasks
   - Activities linked to research
   - Other activity
9. **If you checked "Other activity", please specify**

3. Activities COVID-19 crisis
   1. Among the activities you mentioned in the previous section, were some directly linked to the COVID-19 crisis?
      - Yes
      - No
   2. If yes, those activities were
      - Voluntary
      - Mandatory
   3. **If you were doing those activities on a voluntary basis, which were your motivations?**
   4. Who has been your direct supervisor most often for your activities that were directly linked to the COVID-19 crisis?
      - Medical doctor
      - Nurse
      - Other health care professional
      - Administrative and management personnel
      - Other
      - I did not have the impression to be supervised
   5. **If you checked "Other", thank you for specifying who was supervising you.**
   6. **What were clinical tasks that you carried out?**
   7. **What were sanitary tasks that you carried out?**
   8. **What were logistic tasks that you carried out?**

9. Have you been offered psychological support by the professionals in the place where you carried out your activities?
   - Yes
   - No
10. **If the professionals offered support, thank you for specifying.**

4. COVID-19 crisis
   1. Have you felt isolation over the past three months?
      - Yes
      - No
   2. Have you set up coping strategies to deal with the changes of the past months?
      - Yes
      - No
   3. **If you set up coping strategies, which ones? (Describe even those that seem trivial to you)**
   4. During the past three months, have you felt that you were safe in your place of activity (internship, study, etc.)?
      - Yes
      - No
   5. **If you did not feel safe, thank you for specifying.**
   6. Have you been fearing for your own health during the past three months?
      - Yes
      - No
   7. Did the University of Geneva offer psychological support?
      - Yes
      - No
   8. Did your teachers offer psychological support?
      - Yes
      - No
   9. **If your teachers offered support, thank you for specifying.**
   10. **In your opinion, what has changed the most during these last three months for you, for your training, and your professional identity?**

5. My studies
   1. Where do you study most of the time?
      - Library
      - Cafeteria
      - Other location inside the university
      - Location at a hospital structure
      - At my home
      - Other location outside the university
   2. How do you study most of the time?
      - Alone
      - With someone else
      - In a group
   3. Where have you studied most of the time during the past three months?
      - Library
      - Cafeteria
      - Other location inside the university
      - Location at a hospital structure
      - At my home
      - Other location outside the university
   4. Do you think that the environment where you studied had an impact on your learning?
      - Yes
      - No
   5. **If the environment had an impact, at what level? (Give examples)**
   6. Since March, the vast majority of exams have been reorganized, moving from a sanctioning assessment to a compulsory formative assessment. I think it was a good decision:
      - Totally disagree
      - Rather disagree

- Rather agree
- Totally agree

7. **What changes have been caused for you by the modification of the evaluation format?**
8. Please take position for each of the following statements [Much more often - More often - I did not notice any difference - Less often - Much less often]
    1. For the past three months, I have had difficulty sleeping
    2. For the past three months, I feel sad
    3. For the past three months, I feel anxiety
    4. For the past three months, I manage easily to remain concentrated
    5. For the past three months, I feel tired
    6. For the past three months, I feel full of energy
    7. For the past three months, I feel motivated
    8. For the past three months, I have confidence in myself as a caregiver
    9. For the past three months, relations with relatives living under the same roof have been difficult
    10. For the past three months, relations with relatives not living under the same roof have been difficult
    11. For the past three months, I am concerned by money problems
9. Please take position for each of the following statements [Never - Nearly never - Sometimes - Quite often - Often]
    1. In the past three months, how many times have you been disturbed by an unexpected event?
    2. In the past three months, how many times has it been difficult for you to control the important things in your life?
    3. In the past three months, how often have you felt nervous or stressed?
    4. In the past three months, how often have you felt confident in taking care of your personal problems?
    5. In the past three months, how often have you felt that things were going the way you wanted?
    6. In the past three months, how many times have you felt that you couldn't take on all the things you had to do?
    7. How often in the past three months have you been able to control your nervousness/annoyance?
    8. How often In the past three months have you felt in control of a situation?
    9. In the past three months how often have you felt irritated because things were out of your control?
    10. In the past three months how often have you found that the difficulties got so much that you couldn't control them?

6. Professional identity
    1. Do you have a speciality on mind concerning your future professional career?
        - Yes
        - No
    2. **If yes, which one?**
    3. Did the past three months change your professional perspective?
        - Yes
        - No
    4. **If yes, could you describe this?**
    5. **How would you define your role a future medical doctor?**
    6. Did your vision of this role change over the past three months?
        - Yes
        - No
    7. **If your vision of this role changed, could you describe how?**
    8. Have you felt useful during the past three months?
        - Yes
        - No
    9. Did the feeling of usefulness as future medical doctor evolve over the past three months?
        - Yes
        - No

**10. If yes, how did this feeling change?**

7. Follow up
    1. Would you agree to participate in focus groups?
        - Yes
        - No
    2. Would you agree to be recontacted to participate in interviews?
        - Yes
        - No
    3. Would you agree to be recontacted in the next years to follow up on this study?
        - Yes
        - No
    4. **At what e-mail address or phone number would you like to be contacted?**
